# Geniposidic Acid from *Eucommia ulmoides* Oliver Staminate Flower Tea Mitigates Cellular Oxidative Stress via Activating AKT/NRF2 Signaling

**DOI:** 10.3390/molecules27238568

**Published:** 2022-12-05

**Authors:** Shuo Cheng, Huiling Jia, Yisen Zhang, Juanjuan Zhou, Xue Chen, Lifang Wu, Jun Wang

**Affiliations:** 1 The Center for Ion Beam Bioengineering & Green Agriculture, Hefei Institutes of Physical Science, Chinese Academy of Sciences, Hefei 230031, China; 2 The Science Island Branch of the Graduate School, University of Science and Technology of China, Hefei 230031, China; 3 Zhongke Taihe Experimental Station, Fuyang 236626, China

**Keywords:** *Eucommia ulmoides* Oliver staminate flower tea, oxidative stress, AKT, NRF2, geniposidic acid, keratinocyte

## Abstract

*Eucommia ulmoides* Oliver staminate flower (ESF) tea enjoys a good reputation in folk medicine and displays multiple bioactivities, such as antioxidant and antifatigue properties. However, the underlying biological mechanisms remain largely unknown. In this study, we aimed to investigate whether ESF tea can mitigate cellular oxidative stress. Crude ethyl alcohol extract and its three subfractions prepared by sequential extraction with chloroform, n-butyl alcohol and residual water were prepared from ESF tea. The results of antioxidant activity tests in vitro manifested n-butyl alcohol fraction (n-BUF) showed the strongest antioxidant capacity (DPPH: IC_50_ = 24.45 ± 0.74 μg/mL, ABTS: IC_50_ = 17.25 ± 0.04 μg/mL). Moreover, all subfractions of ESF tea, especially the n-BUF, exhibited an obvious capacity to scavenge the reactive oxygen species (ROS) and stimulate the NRF2 antioxidative response in human keratinocytes HaCaT treated by H_2_O_2_. Using ultra-high-performance liquid chromatography, we identified geniposidic acid (GPA) as the most abundant component in ESF tea extract. Furthermore, it was found that GPA relieved oxidative stress in H_2_O_2_-induced HaCaT cells by activating the Akt/Nrf2/OGG1 pathway. Our findings indicated that ESF tea may be a source of natural antioxidants to protect against skin cell oxidative damage and deserves further development and utilization.

## 1. Introduction

*Eucommia ulmoides* Oliver (EUO) is named Dù-zhòng (in Chinese), Tuchong (in Japanese), as a unique deciduous and dioecious tree, with a long history of medicinal and edible value in east Asia, especially in China, Korea, and Japan [1]. EUO was discussed in chapter 52 of “Shen Ji Zon Lu”, which was composed of theoretical writings and folk medical prescriptions during the Song Dynasty. It was believed to lighten the skin’s pallor and preserve its rejuvenation [2]. Similarly, it was recorded in “Sheng Nong Ben Cao Jing” and “Ben Cao Gang Mu” that the regular consumption of EUO helped people recover easily from fatigue and resist aging [3,4]. These ancient medical records confirmed that EUO was beneficial to the skin.

Additionally, pharmacological studies have demonstrated that the iridoid compounds from the staminate flower of EUO and the methanol extract of the Eucommiae cortex promoted collagen synthesis, which rejuvenated the skin [4,5]. EUO protects skin cells against UVB-induced oxidative stress in vivo and in vitro [6,7]. It was reported that compounds extracted from the different parts (bark, seeds, stem, leaves, and flowers) of EUO contain abundant bioactive substances, such as chlorogenic acid and geniposidic acid. The content of some active substances, such as flavonoids, is higher in the staminate flowers than in its leaves and stems [8]. The blooming period of staminate flowers of *Eucommia ulmoides* is approximately 20 days, and the staminate flower resources are not fully utilized [9,10]. Therefore, previous studies paid more attention to the bark and leaves of EUO, and the biological functions of EUO staminate flowers remain to be investigated [5]. Since the 1980s, the staminate flowers of EUO have been widely used to make tea in China because of their sedative effect and the function of preserving skin rejuvenation [1,5]. Nowadays, the *Eucommia ulmoides* Oliver staminate flower tea (ESF tea), developed using the staminate flower of EUO as raw material, is a popular drink in Asian countries. Its attributes include bright yellow and green color, thick and refreshing taste, distinctive and enduring aroma, as well as beneficial effects on antifatigue and antioxidant activities [10,11,12]. However, the antioxidative effect of ESF tea on the skin and its mechanisms have not been well described.

Skin is the first protective barrier between internal organs and the environment and is more vulnerable to exposure to endogenous and exogenous harmful factors-induced oxidative stress [13,14]. Oxidative stress leads to excessive ROS production, which can cause a series of skin problems, such as atopic dermatitis, acne, melanin deposition, aging, and skin cancer [15,16]. In response to oxidative stress, several antioxidant systems have evolved in organisms to protect them against oxidative damage [17]. For example, the NRF2 antioxidant pathway increases the expression of several redox-balancing proteins and detoxification enzymes through the binding of transcription factor NRF2 to antioxidant stress elements (ARE) sequence, thus improving the antioxidant capacity of the body [18,19]. As a key regulator downstream of the NRF2 signaling pathway, OGG1 is demonstrated to be regulated at the transcriptional level in response to oxidative stress [20]. OGG1 is a bifunctional enzyme with N-glycosylase and β-lyase activities, which can remove abnormal bases to repair DNA oxidative damage [21]. AKT, a critical serine/threonine kinase, can be activated under oxidative stress to promote cell proliferation and survival [22]. Moreover, AKT activates the transcription of antioxidant genes via the Akt-NRF2-ARE pathway [23]. 

Overall, the AKT/NRF2/OGG1 axis is thought to be an important cytoprotective defense mechanism against ROS-induced DNA oxidative damage in cells. In addition to the body’s antioxidant system, the supplement of antioxidants obtained from dietary or exogenous sources is necessary for increasing resistance to oxidative stress and preventing/improving skin diseases [24]. Due to their nontoxic and environmentally friendly qualities, natural compounds with antioxidant activities are preferable to synthetic antioxidants for defending the body against oxidative damage [25,26]. Plant-derived antioxidants with low molecular weight can enter cells quickly, enhancing cellular metabolism, collagen and elastin expression, and antioxidant capacity. These compounds are widely used in skin protection [27,28,29].

In this study, using the human keratinocytes cell line HaCaT, we confirmed that ESF tea extract can attenuate cellular free radical levels. We also found that the ESF tea extract, in particular its major ingredient, geniposide acid, could stimulate the AKT/NRF2/OGG1 antioxidative response and mitigate DNA oxidative damages caused by reactive free radicals. These findings proved that the antioxidative function of ESF tea has potential utilization in applications such as skin protection.

## 2. Results

### 2.1. Total Phenolic and Total Flavonoid Content

Different solvents were used to extract the ESF tea and gather bioactive components. The quantitative results of TPC and TFC of the CE and its subfractions of ESF tea were expressed as mg of gallic acid equivalent per gram (mg GAE/g) and mg of rutin equivalent per gram (mg QE/g), respectively. As Table 1 shows, the n-butanol fraction (272 ± 6.52 mg GAE/g extract) contained the highest total phenolic content, followed by CE (126.4 ± 7.94 mg GAE/g extract), CHF (78.36 ± 4.13 mg GAE/g extract) and RWF (74.13 ± 3.12 mg GAE/g extract). The total flavonoid content was distributed in the following order: n-BUF (286.92 ± 2.56 mg RE/g extract) > CE (124.57 ± 3.56 mg RE/g extract) > CHF (85.3 ± 4.12 mg RE/g extract) > RWF (84.24 ± 2.64 mg RE/g extract). Table 2 revealed a strong positive correlation (r = 0.998) between TPC and TFC. Thus, n-BUF had the maximum TPC and TFC, followed by CE.

### 2.2. Comparison of the In Vitro Antioxidant Activities of Different Fractions of ESF Tea

Antioxidants can contribute electrons to reduce iron ions to ferrous ions; hence, the change in ferrous ions reflects the antioxidant capacity of antioxidants. The reduction capacity was 286.45 ± 11.04 mg of Trolox equivalent per gram in the n-BUF fraction (Table 1). Among the four organic fractions, n-BUF had the strongest ability to reduce iron ions, followed by CE (108.87 ± 2.2 mg Trolox equivalent per gram), RWF (70.42 ± 0.77 mg Trolox equivalent per gram), CHF (47.95 ± 1.32 mg Trolox equivalent per gram). As shown in Table 2, the correlation analysis revealed that TPC and TFC had a significant positive correlation with FRAP values (r = 0.994 and 0.996, respectively). Therefore, we speculated that the content of total phenols and flavonoids was the key factor in determining the substantial variation in the FRAP values between the different extraction fractions.

The DPPH radical has a large absorption peak near 517 nm; however, the antioxidants will react with the DPPH electron pair, weakening its absorption degree. Table 1 shows that the scavenging capacities among the four extract fractions from ESF tea on DPPH were studied. The DPPH scavenging activity of the n-BUF was the highest, which had the lowest IC_50_ (24.45 ± 0.74 μg/mL), followed by CE (59.71 ± 1.78 μg/mL), RWF (101.19 ± 11.23 μg/mL), and CHF (131.68 ± 7.72 μg/mL). Correlation analysis showed that the TPC and TFC values in different fractions were significantly negatively correlated with the IC_50_ values of the DPPH clearance (r = −0.892 and −0.873, respectively).

Table 1 shows that the n-BUF exhibited the strongest scavenging ability of ABTS^+^ radicals with the lowest IC_50_ value of 17.25 ± 0.04 μg/mL, followed by CE (42.78 ± 1.17 μg/mL), CHF (56.52 ± 1.85 μg/mL), and RWF with the highest IC_50_ value (82.94 ± 0.29 μg/mL) exhibited the weakest scavenging ability of ABTS^+^ radical. The ABTS^+^ radical-scavenging activities were in the following order: n-BUF > CE > CHF > RWF. Similar to the results of the DPPH experiment, there was a substantial negative correlation between the TPC and TFC values in different fractions and the IC_50_ values of ABTS^+^ clearance (r = −0.893 and −0.871, respectively). These results demonstrated that the radical clearance capacity (DPPH, ABTS^+^) and reducing power (FRAP) in different fractions from ESF tea were inseparable from the total phenolic and flavonoid content.

### 2.3. Cytoprotective Activity against H_2_O_2_-Induced Oxidative Injury of ESF Tea

**Figure 1:**
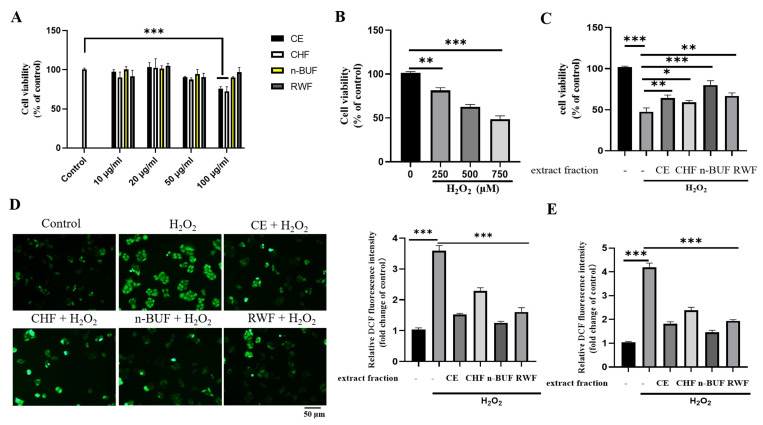
ESF tea ameliorated cytotoxicity and attenuated intracellular ROS generation in H_2_O_2_-treated HaCaT cells. (**A**) HaCaT cells were treated with four extract fractions at the indicated concentrations (10, 20, 50, 100 μg/mL) for 24 h. Cell viability was evaluated by a CCK−8 kit. (**B**) The effect of H_2_O_2_ on human immortalized keratinocyte (HaCaT) cell viability was evaluated by CCK−8. (**C**) HaCaT cells were treated with four extract fractions for 24 h before being exposed to H_2_O_2_ (750 μM). The protective effect of tea extract on cell viability in H_2_O_2_-treated cells was evaluated by CCK−8 kit. (**D**) HaCaT were cultured in 35 mm dishes, treated with different extract fractions (50 μg/mL) for 24 h and then exposed to 750 μM H_2_O_2_. Intracellular ROS generation was measured by a fluorescence microscope using CM−H_2_DCFDA probe. (**E**) Under the same experimental conditions, intracellular ROS intensity was measured with a SpectraMax i3X microplate reader (Molecular Devices, CA, USA) and compared to the control group. Data were represented as mean ± SD of three independent experiments. * *p* < 0.05, ** *p* < 0.01, *** *p* < 0.001.

In this study, the H_2_O_2_-induced oxidative injury of HaCaT cells was used to assess the cytoprotective capabilities of CE and its fractions. As shown in Figure 1A, when the concentrations of extracts ranged from 10 μg/mL to 50 μg/mL, cell viabilities were not significantly affected. We then tested the protection of 50 μg/mL extracts against H_2_O_2_-induced reduction of cell viability. As the H_2_O_2_ concentration rose, cell viability fell rapidly. Based on the significant difference (48.42%) compared to the control group, the optimal concentration of H_2_O_2_ (750 μM) was chosen for the subsequent experiments (Figure 1B). The detrimental effect of H_2_O_2_ on cell viability (47.47%) was attenuated by the ESF tea extract effectively at a concentration of 50 μg/mL (Figure 1C). When pretreatment with different extract fractions (CE, CHF, n-BUF, and RWF), the cell viabilities, increased to 64.27%, 58.88%, 79.89%, and 66.81%, respectively. 

We further detected whether ESF tea extracts could attenuate H_2_O_2_-induced elevation of ROS levels. The ROS scavenging capabilities of different fractions were shown in Figure 1D; the fluorescence intensity of the H_2_O_2_ treatment group alone was around 3.59 times that of the control group, whereas the ROS levels were reduced if HaCaT cells were pretreated with the four fractions before H_2_O_2_ treatment. The n-BUF fraction exhibited the strongest ability to scavenge ROS. The CE and RWF decreased the fluorescence intensity to approximately 1.52 and 1.63 times that of the control group, while the CHF exhibited the weakest intracellular ROS scavenging capacity. Consistent with the aforementioned results, the DCF fluorescence intensity was measured with a microplate reader, revealing that ESF tea extract could effectively reduce ROS production in H_2_O_2_-induced HaCaT cells (Figure 1E). 

**Figure 2:**
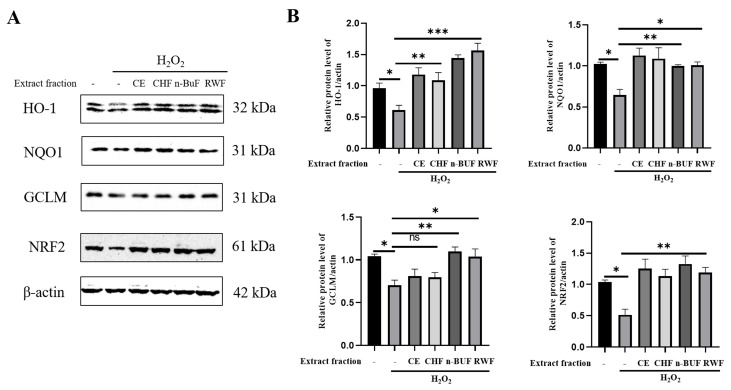
ESF tea protects HaCaT against H_2_O_2_−induced oxidative injury via modulating antioxidative protein expression. Cells were exposed to H_2_O_2_ (750 μM) after being pretreated with four ESF tea extract fractions (50 μg/mL) for 24 h. (**A**) Western blotting was performed to identify the expression level of HO-1, NQO1, GCLM and NRF2. β-actin was used as the internal control. (**B**) The quantitative analysis of the band intensity by ImageJ. * *p* < 0.05, ** *p* < 0.01, *** *p* < 0.001, ns = not significant.

Next, we examined the expression levels of proteins involved in the NRF2 antioxidative response in H_2_O_2_-exposed HaCaT cells pre-treated with ESF tea extracts. As shown in Figure 2, the expression levels of NRF2 and its downstream genes, including HO-1, NQO1, and GCLM, were significantly down-regulated by H_2_O_2_ exposure. However, pretreatment with different extract fractions obviously enhanced the expression of the above four proteins. The group pre-treated with the n-BUF fraction displayed the highest expression levels of these proteins. Our results indicated that the ESF tea extracts stimulated the NRF2 antioxidative pathway in response to H_2_O_2_ exposure.

### 2.4. Qualitative and Quantitative Analyzes of Different Fraction Compounds

**Figure 3:**
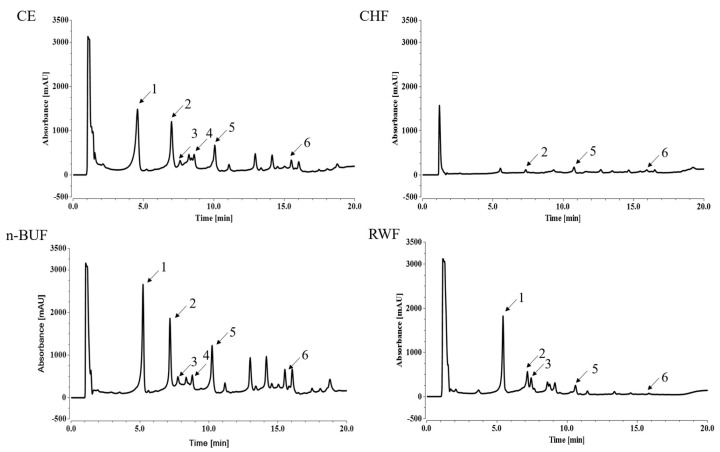
UHPLC chromatograms of four fractions from ESF tea. UV detection at 240 nm. Peak name: 1: geniposidic acid, 2: chlorogenic acid, 3: catechin, 4: caffeic acid, 5: geniposide, 6: rutin.

We next used the UHPLC-DAD method to identify and quantify the dominant compounds in the extract and fractions. As shown in Figure 3, UHPLC-DAD analysis identified six chemical components in different fractions from the ESF tea extract, including geniposidic acid (t_R_ = 5.22 min, peak 1), chlorogenic acid (t_R_ = 7.17 min, peak 2), catechin (t_R_ = 7.75 min, peak 3), caffeic acid (t_R_ = 8.35 min, peak 4), geniposide (t_R_ = 10.23 min, peak 5), and rutin (t_R_ = 12.53 min, peak 6). The quantitative analysis of the compounds was referred to as the calibration curves of standard compounds. The calibration curve with the concentrations of the standards as the abscissa and peak areas of the standards as the ordinate were as follows: geniposidic acid, *Y* = 408.28X + 7.3159 (R^2^ = 0.977); chlorogenic acid, *Y* = 1005.3X − 23.37 (R^2^ = 0.998); catechin, *Y* = 165.41X – 2.33 (R^2^ = 0.998); caffeic acid, *Y* = 1210.8X + 6.27 (R^2^ = 0.955); geniposide, *Y* = 356.37 X + 6.74 (R^2^ = 0.996); and rutin, *Y* = 570.74 X + 4.3817 (R^2^ = 0.993), where *Y* was the peak area, and X was the concentration of the standards, respectively (Figure S1 from Supplementary Materials).

The quantitative results of the main compounds in ESF tea are listed in Table 3 and expressed as mg/g extract. Geniposidic acid was the most abundant in ESF tea. It showed the highest content in n-BUF (148.99 mg/g), which was about 2.5 and 3.9 times to those in CE and RWF, respectively. Chlorogenic acid was found in all four fractions. The amount of chlorogenic acid changed extensively in different fractions and ranged from 1.55 mg/g extract (CHF) to 52.41 mg/g extract (n-BUF). Another iridoid found in ESF tea extract, geniposide, was found to be most abundant in the n-BUF fraction (84.1 mg/g), followed by CE (23.19 mg/g), RWF (3.18 mg/g), and CHF (1.75 mg/g). Because they dissolve more easily in alcohol solutions than in chloroform, catechin and caffeic acid were not present in the CHF fraction. Rutin was mostly concentrated in n-BUF (6.1 mg/g) and CE (4 mg/g), while the content in RWF (0.2 mg/g) was relatively low due to its poor solubility in water in the CHF fraction. Rutin was mostly concentrated in n-BUF (6.1 mg/g) and CE (4 mg/g), while the content in RWF (0.2 mg/g) was relatively low due to its poor solubility in water.

### 2.5. GPA Alleviated H_2_O_2_-Induced Oxidative Stress via AKT/NRF2/OGG1 Signaling Pathway

**Figure 4:**
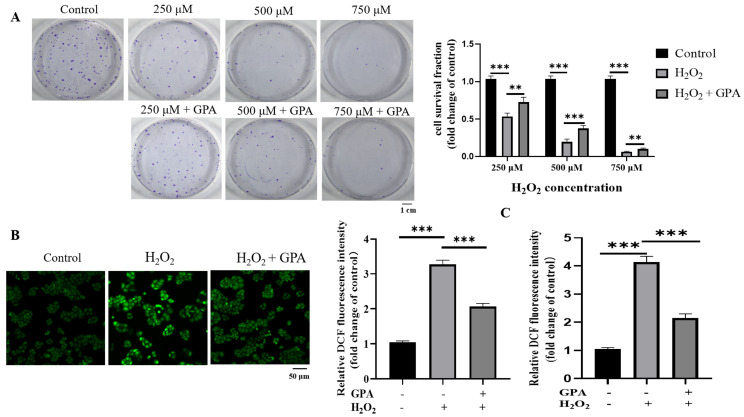

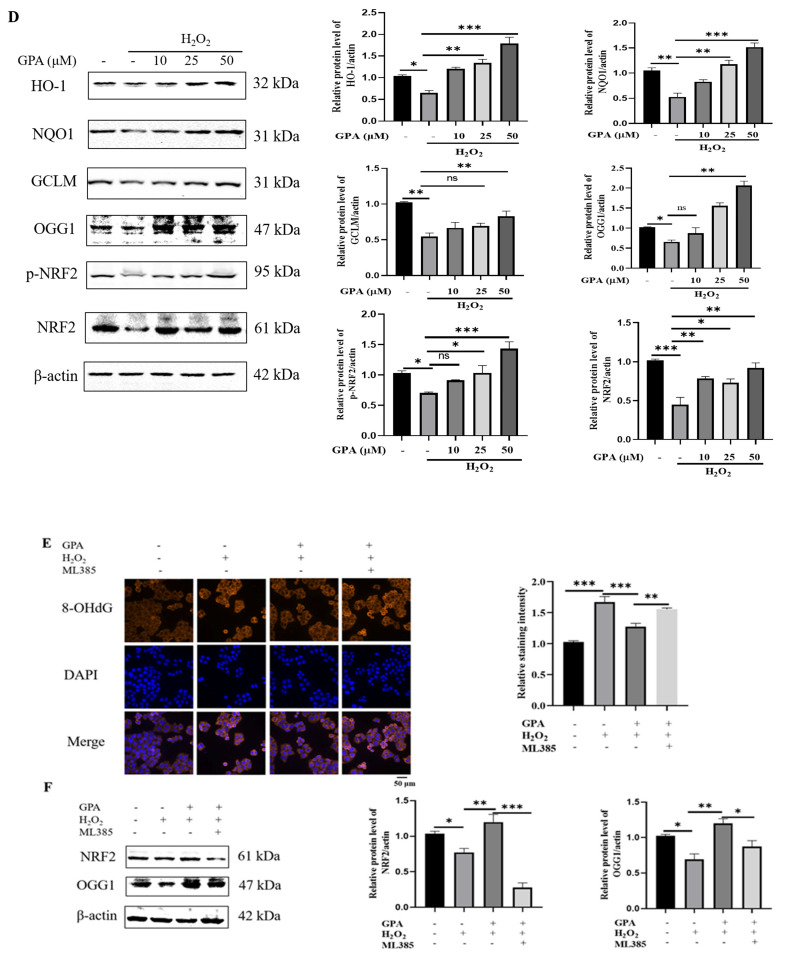
GPA reduced oxidative stress induced by H_2_O_2_ through NRF2/OGG1 signaling. (**A**) HaCaT cells were treated with GPA (50 μM) for 24 h before being exposed to H_2_O_2_ with indicated concentrations (250 μM, 500 μM, 750 μM) for 4 h. Clonogenic surviving assay was used to evaluate the effects of GPA pretreatment on clonogenicity under oxidative stress. (**B**) Cells were exposed to H_2_O_2_ (750 μM) for 4 h after being pretreated with GPA (50 μM) for 24 h. Intracellular ROS generation was measured by a fluorescence microscope using a CM−H_2_DCFDA probe. (**C**) In a parallel experiment, the DCF fluorescence intensity was measured with a SpectraMax i3X microplate reader (Molecular Devices, CA, USA) and compared to the control group. (**D**) Cells were exposed to H_2_O_2_ (750 μM) for 4 h after being pretreated with GPA (10 μM, 25 μM, 50 μM) for 24 h. Western blotting was performed to identify the antioxidative genes expression level. (**E**) The cells were pretreated with GPA (50 μM) for 12 h and then cotreated with ML385 (10 μM) for 12 h before being exposed to H_2_O_2_ (750 μM). After 4 h, the 8-OHdG level of cells was measured using immunofluorescent staining. (**F**) Under the same experimental conditions, the expression levels of NRF2 and OGG1 were determined by Western blotting analysis. * *p* < 0.05, ** *p* < 0.01, *** *p* < 0.001, ns = not significant.

The above result showed that GPA is a dominant ingredient in ESF tea; we then explored whether it contributes to the antioxidative capability of ESF tea. A clonal survival assay was used to evaluate the protection of GPA against H_2_O_2_ treatment-induced HaCaT cell death. As Figure 4A shows, the clonogenic formation rates of HaCaT decreased with the increase in H_2_O_2_ concentration. However, the clonogenic formation rates were increased by pretreatment with GPA. Next, we used 750 µM of H_2_O_2_ to conduct follow-up experiments. We found that similar to the tendencies obtained by using ESF tea extracts, GPA pretreatment effectively decreased the intracellular ROS level induced by H_2_O_2_ (Figure 4B, C) and, in addition, upregulated the expression levels of NRF2 and its downstream antioxidant genes (Figure 4D).

8-OHdG is a pivotal biomarker used to assess DNA oxidative damage levels [30]. Immunofluorescence staining of 8-OHdG was performed to study the effect of GPA on H_2_O_2_-induced formation of DNA oxidative damage. As shown in Figure 4E, H_2_O_2_ increased the formation of 8-OHdG in HaCaT cells by 1.67 times compared with the control group, while GPA pretreatment (50 μM) reduced the level of 8-OHdG by 40%. OGG1 is the primary enzyme in DNA oxidative damage repair pathway, which removes 8-OHdG from DNA induced by ROS [31]. OGG1 expression was inhibited in HaCaT cells exposed to H_2_O_2_, while treatment with GPA (10, 25 and 50 μM) before H_2_O_2_ exposure enhanced the expression of OGG1 to a level around 0.88, 1.56, 2.06 folds, respectively, compared to that in the control group (Figure 4D). It has been reported that NRF2 can activate OGG1 transcription, thereby alleviating oxidative DNA damage [32]. Our results above showed GPA pretreatment stimulated the expression of NRF2 in HaCaT exposed to H_2_O_2_. ML385 is a specific inhibitor of NRF2, which can effectively inhibit the expression of NRF2 in HaCaT cells (Figure S2A from Supplementary Materials). By using ML385, we further confirmed that the increased expression of OGG1 in cells pretreated with GPA was related to NRF2, as evidenced by the fact that the addition of ML385 inhibited GPA-induced OGG1 expression in H_2_O_2_-treated cells (Figure 4F). Meanwhile, ML385 increased H_2_O_2_-induced 8-OHdG formation in GPA-pretreated cells (Figure 4E). These results indicated that NRF2/OGG1 aix contributed to the function of GPA in mitigating H_2_O_2_-induced oxidative damage to cells.

**Figure 5:**
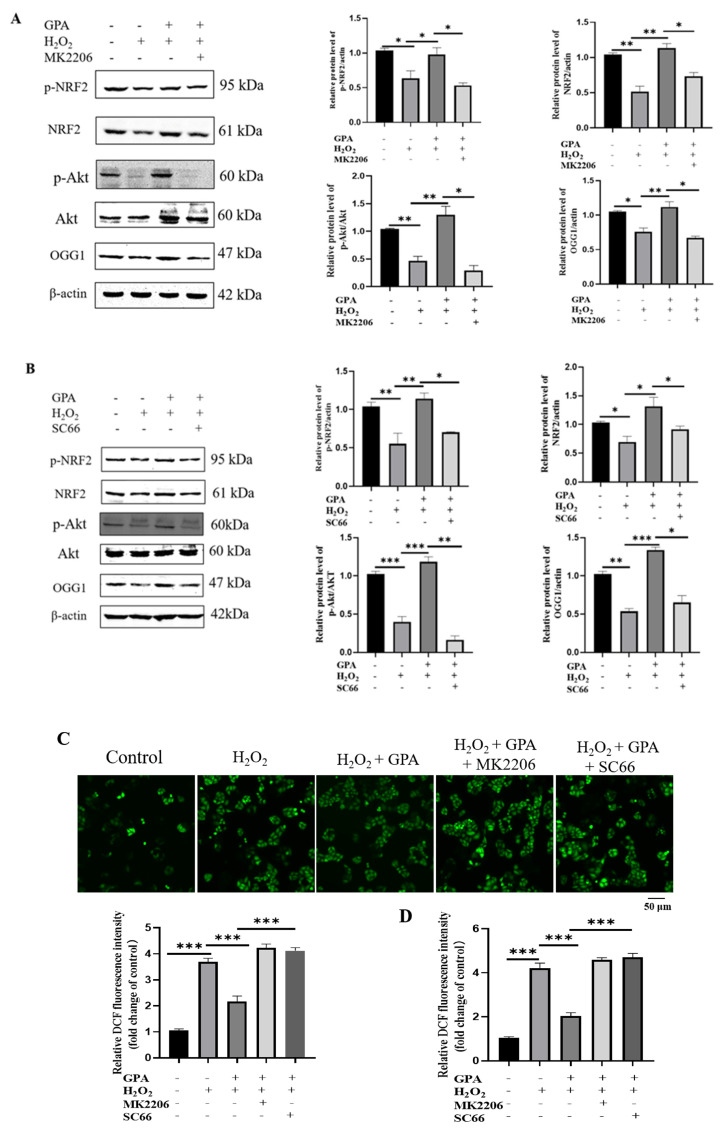
Effects of GPA and inhibition of AKT on phosphorylation of AKT and expression level of NRF2 and its downstream genes. (**A**) Cells were co-pretreated with GPA (50 μM) and MK2206 (10 μM) for 24 h and then exposed to 750 μM H_2_O_2_ for 4 h. Western blotting was performed to identify the corresponding genes’ expression levels. (**B**) HaCaT cells were pretreated with GPA (50 μM), and SC66 (5 μM) for 24 h, followed by treatment with H_2_O_2_ (750 μM) for another 4 h. Cell lysates were collected and then used for Western blotting analysis. (**C**) Under the same experimental conditions, intracellular ROS generation was measured by a fluorescence microscope using a CM−H_2_DCFDA probe. (**D**) In a parallel experiment, the DCF fluorescence intensity was measured with a SpectraMax i3X microplate reader (Molecular Devices, CA, USA) and compared to the control group. * *p* < 0.05, ** *p* < 0.01, *** *p* < 0.001.

Phosphorylation of Ser-40 in NRF2 in response to oxidative stress resulted in its dissociation from its repressor KEAP1 and then promoted its antioxidative function [33]. We found that GPA restored the p-NRF2 (ser-40) expression level in H_2_O_2_-treated HaCaT cells (Figure 4D). Furthermore, AKT-specific inhibitor MK2206 or SC66 was used to verify whether the activation of AKT was involved in the cytoprotective function of GPA. In the presence of MK2206 or SC66, the phosphorylated AKT (ser 473) level in cells was inhibited (Figure S2B,C from Supplementary Materials). As shown in Figure 5A,B, compared to the untreated cells, H_2_O_2_ treatment diminished the p-AKT level, while the GPA pretreatment restored the phosphorylation of AKT. Then, it was found that the induction of p-NRF2 (ser40) by GPA pretreatment in H_2_O_2_-induced HaCaT cells was repressed in the presence of AKT-specific inhibitor MK2206 or SC66, indicating the activation of NRF2 antioxidative response was partially due to the activation of AKT. Additionally, it was found that MK2206 or SC66 treatment inhibited the expression levels of NRF2 and OGG1 in GPA-pretreated cells. As shown in Figure 5C,D, AKT activity inhibition significantly inhibited GPA’s effect in scavenging H_2_O_2_-induced ROS in HaCaT cells. On the whole, these results indicated that GPA pretreatment activated the AKT/NRF2/OGG1 signaling, which protected cells against H_2_O_2_ treatment by alleviating DNA oxidative damage.

## 3. Discussion

Keratinocytes, the most abundant cells in the skin, protect the skin against endogenous and environmental stress by facilitating wound healing and danger signal molecular transduction [34]. The primary ROS, H_2_O_2,_ will accumulate in large quantities in oxidative phosphorylation homeostasis imbalance, ultraviolet light, and environmental pollution, causing skin issues [17,35]. In light of the aforementioned findings, the model of keratinocytes damaged by H_2_O_2_ has been widely established to search for active substances against oxidative skin injury and related mechanisms. In this study, we found that ESF tea extract and its main active compound, GPA, protected HaCaT cells from H_2_O_2_ injury.

We initially extracted *Eucommia ulmoides* Oliver staminate flower tea with ethanol rather than water, then utilized fractional extraction to produce four distinct extracts. There are pieces of evidence to suggest that active plant compounds like flavonoids and phenols may be more soluble in alcohol solutions. For instance, 60% ethanol extract from *Eucommia ulmoides* leaves had higher total phenol and flavonoid levels and a larger antioxidant potential when compared to water extract [36]. An earlier investigation found that antioxidants in tea prepared from *Malus toringoides* leaves are more likely to dissolve in medium-polarity organic solvents [37]. Reynertson et al. discovered that an extract could be divided into four grades owing to its antioxidant capacity (IC_50_ value): high activity (IC_50_ < 50 µg/mL); general activity (50–100 µg/mL); mild activity (100–200 µg/mL); and inactivity (IC_50_ > 200 µg/mL) [38]. Hence, four different fractions from ESF tea demonstrated good radical scavenging and reduction activities, particularly n-BUF. In addition, the n-butanol fraction exhibited the greatest ROS scavenging and antioxidant gene expression increase ability in H_2_O_2_-induced HaCaT cells.

As reported previously, there is a positive correlation between the antioxidant capacity of plant extracts and their phenolic and flavonoid levels [39,40]. Similar to these earlier findings, in this study, the UHPLC analysis results revealed that n-butanol fractions had higher concentrations of active compounds, such as phenols, flavonoids, and iridoids, than did other fractions. Furthermore, we discovered that GPA was substantially more abundant in the four fractions than in the other active substances. It has been reported that geniposidic acid is the active ingredient with high content in EUO. For instance, Wang et al. discovered that the active ingredient in *Eucommia ulmoides* leaf and bark extracts had the highest amount of geniposidic acid [41]. The content of geniposidic acid in staminate flowers and flower tea from EUO was higher than that of chlorogenic acid and geniposide [42]. We suggest that GPA may play an important role in the antioxidant activity of ESF tea. The previous study found that GPA protected the liver against D-galactosamine and lipopolysaccharide-induced hepatic failure in mice through increased NRF2/HO-1 expression levels [43]. Wang et al. discovered that geniposidic acid and its derivative could prolong the replicative lifespan of K6001 yeast by reducing oxidative stress and inducing autophagy [44]. In this study, we found that GPA could efficiently reduce ROS while improving the viability of cell proliferation in H_2_O_2_-induced cells.

NRF2, a redox-sensitive transcription factor that stimulates the expression of antioxidant genes like HO-1, NQO1, and GCLM, serves as the master switch of redox in vivo [45]. Heme oxygenase-1 (HO-1) plays an important role in catalyzing the decomposition of heme oxygenase-1 into carbon monoxide, ferrous iron, and biliverdin [46]. NQO1 can facilitate double electron reduction, protecting biomolecules from oxidative damage brought on by environmental stress [47]. GCLM is involved in the subpathway of glutathione synthesis by L-cysteine and L-glutamate [48]. These antioxidant genes protect cells from oxidative damage and then maintain cell homeostasis. We discovered that ESF tea and its major compound, GPA, relieved oxidative stress in H_2_O_2_-induced HaCaT cells by inducing the expression of NRF2 and its downstream genes. GPA promoted cell survival under oxidative stress and maintained intracellular ROS homeostasis, which may be closely related to the activation of the NRF2 pathway.

Supraphysiological concentrations of reactive oxygen species can directly or indirectly attack biological macromolecules, such as proteins, lipids, and DNA, which trigger inflammation, aging, and cancer [49]. For example, the DNA oxidative damage induced by UV radiation contains cyclobutene pyrimidine dimers (CPDs), pyrimidine (6-4) pyrimidone photodimers, and 8-hydroxy-2′-deoxyguanosine (8-OHdG), resulting in apoptosis of keratinocytes in the epidermis [50]. The biomarker 8-OHdG is frequently used to evaluate oxidative stress and carcinogenesis. OGG1, as a DNA glycosylase, plays an important role in the base excision repair pathway of DNA oxidative damage. It is known that the human OGG1 promoter has ARE sequence, that is, NRF2 binding sites, which are activated in an aberrant redox state [51]. A previous study found that NRF2 silencing inhibited the increase of OGG1 expression induced by phloroglucinol in UVB-irritated HaCaT cells [52].

In our study, H_2_O_2_ stimulation of HaCaT decreased the OGG1 expression level, thereby increasing intracellular 8-OHdG formation. Furthermore, we observed that inhibition of NRF2 activity would counteract the repair effect of GPA on DNA oxidative damage. Although NRF2 activity is regulated by various mechanisms, phosphorylation modification by kinases is crucial for regulating NRF2 activity. For instance, GSK3 decreases NRF2 activity while PKC, PI3K/AKT, and AMPK kinase promote it [53]. Caffeoylserotonin protects HaCaT against H_2_O_2_ injury by upregulating NRF2/HO-1 via AKT activation [54]. Similarly, MK2206 treatment inhibited the activation of NRF2 and the upregulation of OGG1 in HaCaT cells cotreated with GPA and H_2_O_2_. In conclusion, oxidative damage caused by H_2_O_2_ in HaCaT cells could be relieved by GPA through AKT/NRF2/OGG1 pathway.

With the growing demands for functional foods and the increasing interest in effective natural antioxidants, it makes sense for us to perform qualitative and quantitative studies on ESF tea. Our study provides evidence and solid data that ESF tea can be a natural source of antioxidants to protect skin from oxidative damage. Additionally, our work will promote the further development of *Eucommia ulmoides* Oliver staminate flower resources and avoid the waste of valuable staminate flower resources. However, to achieve this goal, further research needs to be done, such as determining the molecular targets of the single isolated molecule from ESF tea, as well as assessing the toxicity and biosafety of ESF tea in clinical investigations.

## 4. Materials and Methods

### 4.1. Chemicals and Reagents

Folin–Ciocalteu reagent, Trolox, TPTZ, DPPH, ABTS, and H_2_O_2_ were acquired from Sigma-Aldrich (Shanghai, China). Authentic standards of compounds for UHPLC-DAD analysis were purchased from Chengdu Purechem-Standard Co., Ltd. (Chengdu, China). GPA used for the cell experiment was acquired from MedChemExpress (≥98%; 27741-01-1; Monmouth Junction, NJ, USA). CCK8 cell viability testing kit was acquired from (GK10001; GlpBio, CA, USA). ROS probes CM-H_2_DCFDA were acquired from (C6827; Invitrogen, CA, USA). ESF tea was purchased from Fangjie Agricultural Development Co., LTD (Lingbao, China). ML385 (99.72%; HY-100523), MK2206 (99.92%; HY-108232), and SC66 (99.82%; HY-19832) were purchased from MedChemExpress (Monmouth Junction, NJ, USA).

### 4.2. Sequential Extraction Process

The ESF tea extract preparation procedures utilized in this study were referred to as that reported by Fan et al. with a slight modification [37]. Briefly, the ESF tea was crushed into powder. An amount of 100 g of powder was degreased three times with petroleum (1000 mL) and then extracted three times with 70% ethanol (1000 mL) for ultrasonic-assisted extraction (200 W) at room temperature for 30 min. For every 100 g of tea powder, 22.49 g of ethanol crude extract (CE) was produced after extraction, filtration, rotary evaporation, and freeze-drying. 18 g of ethanol crude extract was dissolved in water, followed by extraction with chloroform, n-butanol, and water, using liquid–liquid partition (*v/v* was 1:1). Each solvent was extracted thrice. The treatment of different organic fraction extraction liquids was the same as that of the ethanol crude extract, and the final yields of the CHF, n-BUF, and RWF were 3.91, 6.37, and 7.11 g, respectively. All of the samples were stored at −20 °C in the dark.

### 4.3. Total Phenolics and Total Flavonoids Content

The colorimetric method measured the total phenolic content (TPC) of CE and its three subfractions from ESF tea. Briefly, the extract was dissolved in 80% methanol and then adjusted to a final concentration of 10 mg/mL. The reaction mixture contained 10 μL of sample solution, 100 μL of Folin–Ciocalteu reagent, 400 μL of Na_2_CO_3_ solution (15% *m*/*v*), and 1490 μL distilled water. The reaction took place at 37 °C under dark for two hours. The mass concentration of gallic acid was used as the abscissa on a standard curve, and the ordinate was the matching absorbance measured at 765 nm using a spectrophotometer (UV-2550, Shimadzu corporation, Kyoto, Japan).

The content of total flavonoid content (TFC) was measured according to the colorimetric method [55]. The reaction system of 2 mL consisted of 40 μL of sample solution, 60 μL NaNO_2_ (0.4 mg/mL), 60 μL Al (NO_3_)_3_ (10% *m*/*v*), 0.8 mL of NaOH (4% *m*/*v*) and appropriate distilled water. The reaction took place at 37 °C under dark conditions for half an hour. The mass concentration of rutin was used as the abscissa on a standard curve, and the ordinate was the matching absorbance measured at 510 nm using a spectrophotometer.

### 4.4. Antioxidant Activity

The FRAP working solution, which contains acetate buffer, TPTZ, and Fe_2_(SO4)_3_·7H_2_O, was mixed with various concentrations of the ESF tea extracts (1 mL each). The whole reaction took place at 37 °C under dark conditions for 10 min. The reduced ability of the sample to ferrous ions was determined by measuring the absorbance of the reaction product at 593 nm.

The DPPH solution (0.2 mM) was mixed with various concentrations of the ESF tea extracts (1 mL each). The whole reaction took place at 37 °C under dark conditions for 30 min. The ability of the sample to scavenge DPPH radicals was determined by measuring the absorbance of the reaction product at 517 nm. A linear curve with mass concentration as the abscissa and clearance as the ordinate was established to determine the extract concentration, which provides 50% of the DPPH scavenging capacity (IC_50_). We estimated the scavenging capacity of DPPH by following the formula: DPPH scavenging effect (%) = A_1_ − A_2_/A_1_ × 100. What needs to be stated here is that A_1_ represented the reference absorbance and A_2_ represented the sample absorbance.

ABTS (7.4 mmol/L) was mixed with K_2_S_2_O_8_ (2.6 mmol/L) overnight and then diluted 10 times with PBS to obtain the ABTS^+^ working solution. The ABTS^+^ working solution was mixed with various concentrations of the ESF tea extracts (1 mL each). The reaction took place at 37 °C under dark conditions for 30 minutes. A linear curve with mass concentration as the abscissa and clearance as the ordinate was established to determine the extract concentration, which provides 50% of the ABTS^+^ scavenging capacity (IC_50_).

### 4.5. Cell Culture and Viability Tests

Human immortalized HaCaT keratinocytes were purchased from Gaining Biological (Shanghai, China) and cultured in high-glucose Dulbecco’s Modified Eagle’s Medium with 10% fetal bovine serum and 1% antibiotics. Cells were grown in a humid incubator with a constant temperature of 37 °C and 5% CO_2_. After pretreatment with different extract fractions (50 μg/mL) for 24 h, cells were treated with H_2_O_2_ for another 4 h. The viability of HaCaT cells treated with the ESF tea extracts or H_2_O_2_ was determined by the CCK-8 method by measuring the absorbance value of the reaction product at 450 nm using a microplate reader (PERLONG 9602, Beijing, China).

### 4.6. Analysis of Intracellular ROS

A fluorescence probe (CM-H_2_DCFDA) was used to measure the ROS content in HaCaT cells. Briefly, HaCaT cells were cultured in 35 mm dishes and treated for 24 h. Then the cells were treated with 750 μM H_2_O_2_ for 4 h. After washing with PBS, the cells were loaded with 5 μM ROS probe in the incubator. Fluorescence images were taken on an Olympus IX83 fluorescence microscope. Fluorescence intensity was analyzed with ImageJ 1.8.1 software (National Institute of Health, Bethesda, MD, USA). In a parallel experiment, cells were planted onto a light-tight 96-well plate (BS-MP-96W, Biosharp Company, Hefei, China) with a density of 5 × 10^3^ cells per well. The cell treatment condition and ROS probe loading were the same as above. Intracellular ROS production was measured with a SpectraMax i3X microplate reader (Molecular Devices, CA, USA) at 492 nm excitation wavelength and 525 nm emission wavelength.

### 4.7. Western Blotting Analysis

HaCaT cells were treated with four extract fractions of the same concentrations for 24 h before being exposed to 750 μM H_2_O_2_ for 4 h. The treated cells were washed with PBS three times, and an appropriate amount of RIPA lysate containing protease inhibitors was added to obtain the whole cell lysate. Different groups of protein samples were quantified with a BCA kit (Sangon Biotech, Shanghai, China) concerning the standard curve of BSA. Protein samples after boiling denaturation were resolved using 8–12% SDS-PAGE and transferred onto a polyvinylidene fluoride (PVDF) membrane (Roche Diagnostics GmbH; Mannheim, Germany). PVDF membrane was blocked with TBST containing 5% skim milk at room temperature for 1–2 h and then incubated with the primary antibody on a 4 °C shaking table overnight. The membrane was washed three times with TBST to remove the excessive nonspecific binding, followed by incubation with a secondary antibody (Jackson ImmunoResearch Laboratories, Inc; West Grove, PA, USA) on a shaking table at room temperature for 2 h. An Enhanced chemiluminescent substrate method (Boster Biological Technology, Ltd., Wuhan, China) was used to visualize the protein bands in the PVDF membrane, and the relative quantitative analysis of protein expression levels was performed by ImageJ software. The following antibodies were used in this study: HO-1 (1:2000; 10701-1-AP; ProteinTech, Wuhan, China), HO-1 (1:2000; PA5-27338; Invitrogen, USA), NQO1(1:5000; EPR3309; Abcam, Cambridge, UK), GCLM (1:5000; ab126704; Abcam, Cambridge, UK), Phospho-NRF2 (1:1000; AJ15556; Abgent, San Diego, CA, USA), NRF2 (1:1000; R1312-8; Huabio, Hangzhou, China), AKT (1:1000; CY5551; Abways, China), β-actin (1:20000; AB0033; Abways, Shanghai, China), p-AKT (1:1000; 4060; Cell Signal Technology, MA, USA), and OGG1 (1:1000; 15125-1-AP; ProteinTech, Wuhan, China).

### 4.8. UHPLC-DAD Analysis

The main chemical constituents of ESF tea extracts were confirmed by Ultra-High Performance Liquid Chromatography (UHPLC)-DAD system equipped with a reverse-phase water acquity C_18_ column (2.1 × 100 mm, 1.7 μm). The entire mobile phase procedure lasted for 20 min, and the specific elution gradient was as follows: 0–3 min, 3% B; 3–6 min, 10% B; 6–15 min, 10–20% B; 15–17 min, 20–55% B; and 17–20 min, 3% B. Where A was acetic acid in water at 0.04% (*v/v*), B was acetonitrile. 3.0 μL of injection volume was used, and the system was run at 30 °C with a flow rate of 0.3 mL/min. Data were recorded at 240 nm. The main compounds in the different fractions were identified after comparing the retention times and UV spectra of pure standard and samples. Samples and standards were dissolved in HPLC grade 75% methanol. Before beginning the test, all samples and standards were filtered through a 0.22 μM filter membrane to remove particulate matter and centrifuged to eliminate bubbles.

### 4.9. Clonogenic Surviving Assay

The cells were pretreated with GPA (50 μM) for 24 h and then exposed to different concentrations of H_2_O_2_ for 4 h. They were then trypsinized, counted, and seeded into 60 mm dishes (400 cells/dish). After 14 days of incubation in an incubator, cells were washed with PBS and then fixed with methanol and acetic acid (9:1, *v/v*). Finally, the fixed cells were stained with crystal violet for 30 min. The colonies containing over 50 cells were counted with the help of a microscope.

### 4.10. Immunofluorescence Staining

To assess the levels of oxidative DNA damage, HaCaT cells were grown on glass coverslips. After washing with PBS three times, the cells were fixed with 4% paraformaldehyde (pH 7.4). The cells were then treated with 0.3% Triton-X100 for 20 min to increase membrane permeability. Subsequently, the cells were blocked with 1% BSA in PBS (0.1% Triton X-100) at 37 °C for 2 h. Next, the cells were incubated with 8-OHdG antibody (1:100; sc-66036; Santa Cruz, CA, USA) at 4 °C overnight. After rinsing with PBS three times, cells were incubated with Alexa Fluor-594-inked secondary antibody for 2 h at room temperature. DAPI was used to counterstain cell nuclei. Fluorescence images were acquired under a fluorescence microscope and analyzed with ImageJ software.

### 4.11. Statistical Analysis

All statistical data were expressed as the mean ± standard deviation based on three repeated experiments. *p* < 0.05 was considered a significant difference, and significance analysis was performed using GraphPad Prism 8. The significant differences among multiple groups were analyzed by one-way ANOVA, followed by the least significant difference (LSD) tests.

## 5. Conclusions

**Figure 6:**
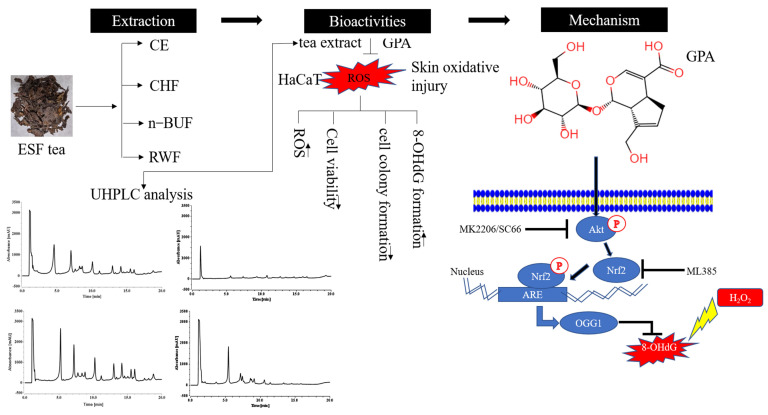
The schematic figure of this study. CE and its three subfractions were prepared by sequential extraction from ESF tea. The active components and antioxidant function were compared among four fractions. As the main active component in ESF tea extract, GPA protected cells against oxidative stress via the AKT/NRF2/OGG1 pathway.

In this study, we found that n-BUF had the highest TPC and TFC and showed the most potent antioxidant properties among the four fractions. The UHPLC analysis demonstrated that most compounds were enriched in n-BUF, and geniposidic acid was the most abundant component in ESF tea. We confirmed that GPA might be the key contributor to the antioxidative capability of ESF tea, which activated the AKT/NRF2 signaling in HaCaT treated by H_2_O_2_. Our findings suggest that ESF tea can be used as a potential source of antioxidants in skin protection (Figure 6).

## Figures and Tables

**Table 1:** Total phenolic and flavonoid content and antioxidant capacity of four fractions from ESF tea.

Sample	TPC	TFC	FRAP	DPPH		ABTS	
	mg GAE/g	mg RE/g	mg Trolox/g	IC_50_ μg/mL	mg Trolox/g	IC_50_ μg/mL	mg Trolox/g
CE	126 ± 7.94 ^b^	124.57 ± 3.56 ^b^	108.87 ± 2.2 ^b^	59.71 ± 1.78	58.97 ± 6.29 ^b^	42.78 ± 1.17	284.27 ± 45.38 ^b^
CHF	78.36 ± 4.13 ^c^	85.3 ± 4.12 ^c^	47.95 ± 1.32 ^d^	131.68 ± 7.72	26.83 ± 3.60 ^c^	56.52 ± 1.85	215.27 ± 35.58 ^b^
n-BUF	272 ± 6.52 ^a^	286.92 ± 2.56 ^a^	286.45 ± 11.04 ^a^	24.45 ± 0.74	144.04 ± 15.26 ^a^	17.25 ± 0.04	703.21 ± 94.90 ^a^
RWF	74.13 ± 3.12 ^c^	84.24 ± 2.64 ^c^	70.42 ± 0.77 ^c^	101.19 ± 11.23	34.81 ± 1.45 ^c^	82.94 ± 0.29	146.31 ± 19.92 ^b^

CE, CHF, n-BuF, and RWF represent ethyl alcohol crude extract, chloroform fraction, n-butanol fraction, and residual water fraction, respectively. In each column, different letters (a,b,c,d) indicate significant differences (*p* < 0.05). Meanwhile, all the results were expressed as the equivalent of the corresponding standard reference [mg gallic acid equivalent per gram (mg GAE/g); and mg rutin equivalent per gram (mg QE/g); mg Trolox equivalent per gram (mg Trolox/g)].

**Table 2:** Correlation between the phenolic and flavonoid content and the antioxidant activities of ESF tea.

	TPC	TFC	FRAP	DPPH	ABTS	IC_50_	
						DPPH	ABTS
TPC	1	0.998	0.994	0.997	0.994	−0.893	−0.893
TFC		1	0.996	0.996	0.994	−0.873	−0.871

**Table 3:** Quantitation of major chemical compounds in different extract fractions by the UHPLC-DAD method.

Compounds	Molecular Formula	CE (mg/g)	CHF (mg/g)	n-BUF (mg/g)	RWF (mg/g)
Geniposidic acid	C_16_H_22_O_10_	60.65 ± 0.01	-	149.0 ± 3.83	37.75 ± 0.92
Chlorogenic acid	C_16_H_18_O_9_	41.69 ± 7.67	1.55 ± 0.61	52.41 ± 0.63	5.53 ± 1.45
Catechin	C_15_H_14_O_6_	5.09 ± 0.3	-	13.34 ± 0.31	1.03 ± 0.26
Caffeic acid	C_9_H_8_O_4_	0.29 ± 0.01	-	2.4 ± 0.2	-
Geniposide	C_17_H_24_O_10_	23.19 ± 0.19	1.67 ± 0.23	84.10 ± 4.9	3.18 ± 0.25
Rutin	C_27_H_30_O_16_	4.003 ± 0.03	0.25 ± 0.03	12.66 ± 0.64	0.43 ± 0.03

## Data Availability

Not applicable.

## Supplementary Materials

The following supporting information can be downloaded at: https://www.mdpi.com/article/10.3390/molecules27238568/s1, Figure S1: The standard curve of six pure compounds. Figure S2: Effects of inhibitors on keratinocytes.
