# Supplementary material for: Geniposidic Acid from Eucommia ulmoides Oliver Staminate Flower Tea Mitigates Cellular Oxidative Stress via Activating AKT/NRF2 Signaling
Source: Molecules. 2022 Dec 5;27(23):8568. doi: 10.3390/molecules27238568 (PMC9739628; doi:10.3390/molecules27238568)
Supplement: Supplementary file 1 [file molecules-27-08568-s001.zip › molecules-2025569-supplementary.pdf]

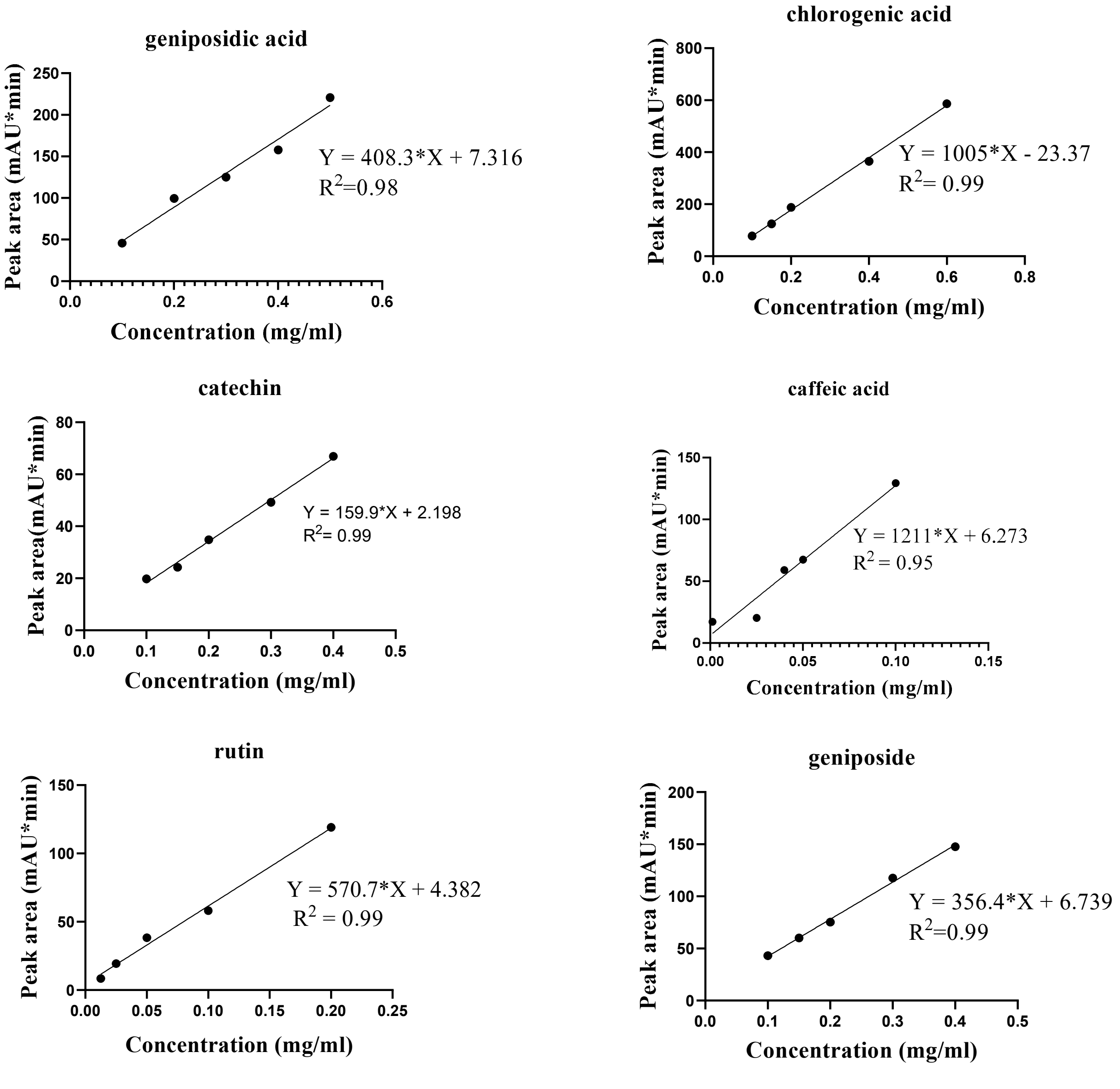

Figure S1: The standard curve of six pure compounds.

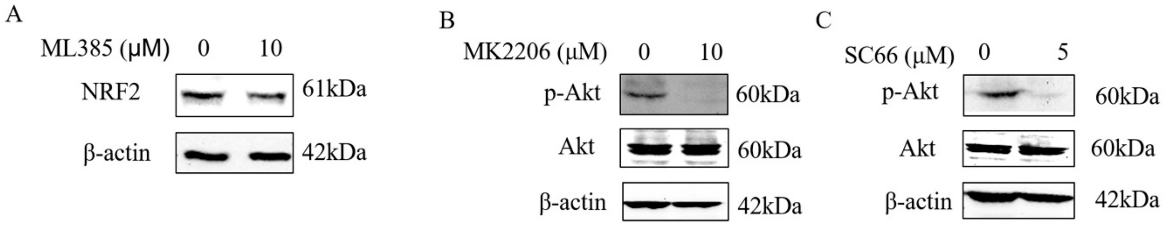

Figure S2. Effects of inhibitors on keratinocytes. (A) Cells were treated with ML385 (10 μM) for 12 h. Western blotting was performed to identify the expression level of NRF2. β-actin was used as the internal control. (B) Cells were treated with MK2206 (10 μM) for 24 h. Western blotting was performed to identify the expression level of p-AKT and AKT. (C) Cells were treated with SC66 (5 μM) for 24 h. Western blotting was performed to identify the expression level of p-AKT and AKT.
